# Supplementary material for: Drought Stress Responses in Context-Specific Genome-Scale Metabolic Models of Arabidopsis thaliana
Source: Metabolites. 2020 Apr 18;10(4):159. doi: 10.3390/metabo10040159 (PMC7241242; doi:10.3390/metabo10040159)
Supplement: Supplementary file 1 [file metabolites-10-00159-s001.zip › metabolites-736140-SP/Supplementary data 2.pdf]

Supplementary data 2

Biomass production reactions in the 26 context-specific GEMs.

Coefficient	Metabolites
-0.04	4-Coumaryl alcohol_biomass
30	ADP_c
-0.0029	ATP_biomass
-30	ATP_c
-0.0033	CTP_biomass
-0.305	Cellulose_biomass
-0.048	Coniferyl alcohol_biomass
-0.282	D-Xylose_biomass
-0.0039	GTP_biomass
-0.053	Glucose_biomass
-0.14	Glycine_biomass
-0.026	Hexadecanoic acid_biomass
-0.107	L-Alanine_biomass
-0.39	L-Asparagine_biomass
-0.162	L-Aspartate_biomass
-0.024	L-Cysteine_biomass
-0.39	L-Glutamate_biomass
-0.35	L-Glutamine_biomass
-0.06	L-Isoleucine_biomass
-0.14	L-Leucine_biomass
-0.001	L-Lysine_biomass
-0.002	L-Methionine_biomass
-0.08	L-Phenylalanine_biomass
-0.01	L-Proline_biomass
-0.21	L-Serine_biomass
-0.086	L-Threonine_biomass
-0.04	L-Tryptophan_biomass
-0.03	L-Tyrosine_biomass
-0.1	L-Valine_biomass
30	Orthophosphate_c
-0.056	Sinapyl alcohol biomass
-0.265	Starch biomass
-0.096	Sucrose biomass
-0.0029	UTP_biomass
-0.053	beta-D-Fructose_biomass
-0.0027	dATP_biomass
-0.0019	dCTP_biomass
-0.0017	dGTP_biomass
-0.0028	dTTP_biomass
