# Supplementary material for: Drought Stress Responses in Context-Specific Genome-Scale Metabolic Models of Arabidopsis thaliana
Source: Metabolites. 2020 Apr 18;10(4):159. doi: 10.3390/metabo10040159 (PMC7241242; doi:10.3390/metabo10040159)
Supplement: Supplementary file 1 [file metabolites-10-00159-s001.zip › metabolites-736140-SP/Supplementary data 3/The context-specific GEMs.html]

Script for tailoring GEM


# The context-specific AraGEMs¶

In [1]:

```
initCobraToolbox(false) %don't update the toolbox

changeCobraSolver ('gurobi', 'all');
```

```
      _____   _____   _____   _____     _____     |
     /  ___| /  _  \ |  _  \ |  _  \   / ___ \    |   COnstraint-Based Reconstruction and Analysis
     | |     | | | | | |_| | | |_| |  | |___| |   |   The COBRA Toolbox - 2019
     | |     | | | | |  _  { |  _  /  |  ___  |   |
     | |___  | |_| | | |_| | | | \ \  | |   | |   |   Documentation:
     \_____| \_____/ |_____/ |_|  \_\ |_|   |_|   |   http://opencobra.github.io/cobratoolbox
                                                  | 

 > Checking if git is installed ...  Done (version: 2.21.0).
 > Checking if the repository is tracked using git ...  Done.
 > Checking if curl is installed ...  Done.
 > Checking if remote can be reached ...  Done.
 > Initializing and updating submodules (this may take a while)... Done.
 > Adding all the files of The COBRA Toolbox ...  Done.
 > Define CB map output... set to svg.
 > TranslateSBML is installed and working properly.
 > Configuring solver environment variables ...
   - [-*--] ILOG_CPLEX_PATH: /Applications/CPLEX_Studio_Community128/cplex/matlab/x86-64_osx
   - [*---] GUROBI_PATH: /Library/gurobi810/mac64/matlab
   - [----] TOMLAB_PATH: --> set this path manually after installing the solver ( see https://opencobra.github.io/cobratoolbox/docs/solvers.html )
   - [-*--] MOSEK_PATH: /Users/rsiriwach/mosek/8/toolbox/r2014a
   Done.
 > Checking available solvers and solver interfaces ...Warning: File: /Applications/CPLEX_Studio_Community128/cplex/matlab/x86-64_osx/@Cplex/Cplex.p Line: 965 Column: 0
Defining "changedParam" in the nested function shares it with the parent function.  In a future release, to share "changedParam" between parent and nested functions, explicitly define it in the parent function.
> In changeCobraSolver (line 423)
  In initCobraToolbox (line 388)
 Done.
 > Setting default solvers ... Done.
 > Saving the MATLAB path ... Done.
   - The MATLAB path was saved in the default location.

 > Summary of available solvers and solver interfaces

			Support 	   LP 	 MILP 	   QP 	 MIQP 	  NLP
	----------------------------------------------------------------------
	gurobi       	active        	    1 	    1 	    1 	    1 	    -
	ibm_cplex    	active        	    1 	    1 	    1 	    - 	    -
	tomlab_cplex 	active        	    0 	    0 	    0 	    0 	    -
	glpk         	active        	    1 	    1 	    - 	    - 	    -
	mosek        	active        	    1 	    - 	    1 	    - 	    -
	matlab       	active        	    1 	    - 	    - 	    - 	    1
	cplex_direct 	active        	    0 	    0 	    0 	    0 	    -
	dqqMinos     	active        	    1 	    - 	    - 	    - 	    -
	pdco         	active        	    1 	    - 	    1 	    - 	    -
	quadMinos    	active        	    1 	    - 	    - 	    - 	    -
	qpng         	passive       	    - 	    - 	    1 	    - 	    -
	tomlab_snopt 	passive       	    - 	    - 	    - 	    - 	    0
	lp_solve     	legacy        	    1 	    - 	    - 	    - 	    -
	----------------------------------------------------------------------
	Total        	-             	    9 	    3 	    5 	    1 	    1

 + Legend: - = not applicable, 0 = solver not compatible or not installed, 1 = solver installed.


 > You can solve LP problems using: 'gurobi' - 'ibm_cplex' - 'glpk' - 'mosek' - 'matlab' - 'pdco' 
 > You can solve MILP problems using: 'gurobi' - 'ibm_cplex' - 'glpk' 
 > You can solve QP problems using: 'gurobi' - 'ibm_cplex' - 'mosek' - 'pdco' - 'qpng' 
 > You can solve MIQP problems using: 'gurobi' 
 > You can solve NLP problems using: 'matlab' 

> Checking for available updates ... skipped

 > Gurobi interface added to MATLAB path.
 > The compatibility can only be evaluated on the following mac OS versions: macOS 10.12, macOS 10.13.
 > The solver compatibility is not tested with MATLAB R2018b.
 > Solver for LP problems has been set to gurobi.

 > Gurobi interface added to MATLAB path.
 > The compatibility can only be evaluated on the following mac OS versions: macOS 10.12, macOS 10.13.
 > The solver compatibility is not tested with MATLAB R2018b.
 > Solver for MILP problems has been set to gurobi.

 > Gurobi interface added to MATLAB path.
 > The compatibility can only be evaluated on the following mac OS versions: macOS 10.12, macOS 10.13.
 > The solver compatibility is not tested with MATLAB R2018b.
 > Solver for QP problems has been set to gurobi.

 > Gurobi interface added to MATLAB path.
 > The compatibility can only be evaluated on the following mac OS versions: macOS 10.12, macOS 10.13.
 > The solver compatibility is not tested with MATLAB R2018b.
 > Solver for MIQP problems has been set to gurobi.
 > Solver gurobi not supported for problems of type NLP. Currently used: matlab
```

In [2]:

```
%path of AraGEM filename
file = 'AraGEMconstraintFree.xml';
```

In [3]:

```
%read model
model = readCbModel(file);
```

In [4]:

```
%path of transcriptome data
T = readtable('GSE65046_mean.csv');
```

```
Warning: Variable names were modified to make them valid MATLAB identifiers. The original names are saved in the VariableDescriptions property.
```

In [5]:

```
% set model objective function_Biomass production

modelConstrained = model;
modelConstrained.c = 0*modelConstrained.c;
modelConstrained = changeObjective(modelConstrained, 'BIO_L', 1);
```

In [6]:

```
% Extraction model base at quantile 0.83 mimic reported biomass and collect info of active metabolites and active reactions

d = 1;
summaryT = table;
for c = 7:33
    i = T.Properties.VariableNames{c};
    
    %calculate cut-off expression threshold at percentile 83
    sorti = sort(T.(i));
    q = sorti(ceil(0.83*(length(sort(sorti))))); 
    
    %mapping gene of expression data to model 
    expressionData = table(T.ORF,T.(i),'VariableNames',{'gene','value'}); 
    [expressionRxns parsedGPR] = mapExpressionToReactions(model, expressionData); 
    
    %tailor model using GIMME
    tissueModel = GIMME(modelConstrained, expressionRxns, q); 
        
    %FBA with  maximization of biomass
    solution = optimizeCbModel(tissueModel,'max'); 

    
    %Table of summary number of the context-specific GEMs
    x = table({i}, length(tissueModel.mets),length(tissueModel.rxns),solution.f);
    summaryT = [summaryT; x]; 
    
    %construct data structure for mets, rxns details of extracted GEMs
    summaryTissueModel(d).model = tissueModel;
    summaryTissueModel(d).model.modelID = i;
    summaryTissueModel(d).name = i;
    summaryTissueModel(d).mets = tissueModel.mets;
    summaryTissueModel(d).rxns = tissueModel.rxns;
    summaryTissueModel(d).biomassFBA = solution.f;
    summaryTissueModel(d).flux = solution.x;
    summaryTissueModel(d).solution = solution;
    
    i
    writeCbModel(tissueModel, 'fileName', i,'format', 'sbml');
    
    d = d + 1;
    end
    
%add property name    
summaryT.Properties.VariableNames ={'condition','mets','rxns','FBA'};
```

```
i =

    'x0Zero'

Warning: Unknown compartment: C_acc.C_acc can be specified in compSymbolList and compNameList.
> In writeSBML (line 287)
  In writeCbModel (line 218)
Warning: Unknown compartment: C_biomass.C_biomass can be specified in compSymbolList and compNameList.
> In writeSBML (line 287)
  In writeCbModel (line 218)
Warning: Unknown compartment: C_c.C_c can be specified in compSymbolList and compNameList.
> In writeSBML (line 287)
  In writeCbModel (line 218)
Warning: Unknown compartment: C_ext.C_ext can be specified in compSymbolList and compNameList.
> In writeSBML (line 287)
  In writeCbModel (line 218)
Warning: Unknown compartment: C_m.C_m can be specified in compSymbolList and compNameList.
> In writeSBML (line 287)
  In writeCbModel (line 218)
Warning: Unknown compartment: C_p.C_p can be specified in compSymbolList and compNameList.
> In writeSBML (line 287)
  In writeCbModel (line 218)
Warning: Unknown compartment: C_v.C_v can be specified in compSymbolList and compNameList.
> In writeSBML (line 287)
  In writeCbModel (line 218)
Warning: Unknown compartment: C_x.C_x can be specified in compSymbolList and compNameList.
> In writeSBML (line 287)
  In writeCbModel (line 218)
Document written

i =

    'x1Control'

Warning: Unknown compartment: C_acc.C_acc can be specified in compSymbolList and compNameList.
> In writeSBML (line 287)
  In writeCbModel (line 218)
Warning: Unknown compartment: C_biomass.C_biomass can be specified in compSymbolList and compNameList.
> In writeSBML (line 287)
  In writeCbModel (line 218)
Warning: Unknown compartment: C_c.C_c can be specified in compSymbolList and compNameList.
> In writeSBML (line 287)
  In writeCbModel (line 218)
Warning: Unknown compartment: C_ext.C_ext can be specified in compSymbolList and compNameList.
> In writeSBML (line 287)
  In writeCbModel (line 218)
Warning: Unknown compartment: C_m.C_m can be specified in compSymbolList and compNameList.
> In writeSBML (line 287)
  In writeCbModel (line 218)
Warning: Unknown compartment: C_p.C_p can be specified in compSymbolList and compNameList.
> In writeSBML (line 287)
  In writeCbModel (line 218)
Warning: Unknown compartment: C_v.C_v can be specified in compSymbolList and compNameList.
> In writeSBML (line 287)
  In writeCbModel (line 218)
Warning: Unknown compartment: C_x.C_x can be specified in compSymbolList and compNameList.
> In writeSBML (line 287)
  In writeCbModel (line 218)
Document written

i =

    'x2Control'

Warning: Unknown compartment: C_acc.C_acc can be specified in compSymbolList and compNameList.
> In writeSBML (line 287)
  In writeCbModel (line 218)
Warning: Unknown compartment: C_biomass.C_biomass can be specified in compSymbolList and compNameList.
> In writeSBML (line 287)
  In writeCbModel (line 218)
Warning: Unknown compartment: C_c.C_c can be specified in compSymbolList and compNameList.
> In writeSBML (line 287)
  In writeCbModel (line 218)
Warning: Unknown compartment: C_ext.C_ext can be specified in compSymbolList and compNameList.
> In writeSBML (line 287)
  In writeCbModel (line 218)
Warning: Unknown compartment: C_m.C_m can be specified in compSymbolList and compNameList.
> In writeSBML (line 287)
  In writeCbModel (line 218)
Warning: Unknown compartment: C_p.C_p can be specified in compSymbolList and compNameList.
> In writeSBML (line 287)
  In writeCbModel (line 218)
Warning: Unknown compartment: C_v.C_v can be specified in compSymbolList and compNameList.
> In writeSBML (line 287)
  In writeCbModel (line 218)
Warning: Unknown compartment: C_x.C_x can be specified in compSymbolList and compNameList.
> In writeSBML (line 287)
  In writeCbModel (line 218)
Document written

i =

    'x3Control'

Warning: Unknown compartment: C_acc.C_acc can be specified in compSymbolList and compNameList.
> In writeSBML (line 287)
  In writeCbModel (line 218)
Warning: Unknown compartment: C_biomass.C_biomass can be specified in compSymbolList and compNameList.
> In writeSBML (line 287)
  In writeCbModel (line 218)
Warning: Unknown compartment: C_c.C_c can be specified in compSymbolList and compNameList.
> In writeSBML (line 287)
  In writeCbModel (line 218)
Warning: Unknown compartment: C_ext.C_ext can be specified in compSymbolList and compNameList.
> In writeSBML (line 287)
  In writeCbModel (line 218)
Warning: Unknown compartment: C_m.C_m can be specified in compSymbolList and compNameList.
> In writeSBML (line 287)
  In writeCbModel (line 218)
Warning: Unknown compartment: C_p.C_p can be specified in compSymbolList and compNameList.
> In writeSBML (line 287)
  In writeCbModel (line 218)
Warning: Unknown compartment: C_v.C_v can be specified in compSymbolList and compNameList.
> In writeSBML (line 287)
  In writeCbModel (line 218)
Warning: Unknown compartment: C_x.C_x can be specified in compSymbolList and compNameList.
> In writeSBML (line 287)
  In writeCbModel (line 218)
Document written

i =

    'x4Control'

Warning: Unknown compartment: C_acc.C_acc can be specified in compSymbolList and compNameList.
> In writeSBML (line 287)
  In writeCbModel (line 218)
Warning: Unknown compartment: C_biomass.C_biomass can be specified in compSymbolList and compNameList.
> In writeSBML (line 287)
  In writeCbModel (line 218)
Warning: Unknown compartment: C_c.C_c can be specified in compSymbolList and compNameList.
> In writeSBML (line 287)
  In writeCbModel (line 218)
Warning: Unknown compartment: C_ext.C_ext can be specified in compSymbolList and compNameList.
> In writeSBML (line 287)
  In writeCbModel (line 218)
Warning: Unknown compartment: C_m.C_m can be specified in compSymbolList and compNameList.
> In writeSBML (line 287)
  In writeCbModel (line 218)
Warning: Unknown compartment: C_p.C_p can be specified in compSymbolList and compNameList.
> In writeSBML (line 287)
  In writeCbModel (line 218)
Warning: Unknown compartment: C_v.C_v can be specified in compSymbolList and compNameList.
> In writeSBML (line 287)
  In writeCbModel (line 218)
Warning: Unknown compartment: C_x.C_x can be specified in compSymbolList and compNameList.
> In writeSBML (line 287)
  In writeCbModel (line 218)
Document written

i =

    'x5Control'

Warning: Unknown compartment: C_acc.C_acc can be specified in compSymbolList and compNameList.
> In writeSBML (line 287)
  In writeCbModel (line 218)
Warning: Unknown compartment: C_biomass.C_biomass can be specified in compSymbolList and compNameList.
> In writeSBML (line 287)
  In writeCbModel (line 218)
Warning: Unknown compartment: C_c.C_c can be specified in compSymbolList and compNameList.
> In writeSBML (line 287)
  In writeCbModel (line 218)
Warning: Unknown compartment: C_ext.C_ext can be specified in compSymbolList and compNameList.
> In writeSBML (line 287)
  In writeCbModel (line 218)
Warning: Unknown compartment: C_m.C_m can be specified in compSymbolList and compNameList.
> In writeSBML (line 287)
  In writeCbModel (line 218)
Warning: Unknown compartment: C_p.C_p can be specified in compSymbolList and compNameList.
> In writeSBML (line 287)
  In writeCbModel (line 218)
Warning: Unknown compartment: C_v.C_v can be specified in compSymbolList and compNameList.
> In writeSBML (line 287)
  In writeCbModel (line 218)
Warning: Unknown compartment: C_x.C_x can be specified in compSymbolList and compNameList.
> In writeSBML (line 287)
  In writeCbModel (line 218)
Document written

i =

    'x6Control'

Warning: Unknown compartment: C_acc.C_acc can be specified in compSymbolList and compNameList.
> In writeSBML (line 287)
  In writeCbModel (line 218)
Warning: Unknown compartment: C_biomass.C_biomass can be specified in compSymbolList and compNameList.
> In writeSBML (line 287)
  In writeCbModel (line 218)
Warning: Unknown compartment: C_c.C_c can be specified in compSymbolList and compNameList.
> In writeSBML (line 287)
  In writeCbModel (line 218)
Warning: Unknown compartment: C_ext.C_ext can be specified in compSymbolList and compNameList.
> In writeSBML (line 287)
  In writeCbModel (line 218)
Warning: Unknown compartment: C_m.C_m can be specified in compSymbolList and compNameList.
> In writeSBML (line 287)
  In writeCbModel (line 218)
Warning: Unknown compartment: C_p.C_p can be specified in compSymbolList and compNameList.
> In writeSBML (line 287)
  In writeCbModel (line 218)
Warning: Unknown compartment: C_v.C_v can be specified in compSymbolList and compNameList.
> In writeSBML (line 287)
  In writeCbModel (line 218)
Warning: Unknown compartment: C_x.C_x can be specified in compSymbolList and compNameList.
> In writeSBML (line 287)
  In writeCbModel (line 218)
Document written

i =

    'x7Control'

Warning: Unknown compartment: C_acc.C_acc can be specified in compSymbolList and compNameList.
> In writeSBML (line 287)
  In writeCbModel (line 218)
Warning: Unknown compartment: C_biomass.C_biomass can be specified in compSymbolList and compNameList.
> In writeSBML (line 287)
  In writeCbModel (line 218)
Warning: Unknown compartment: C_c.C_c can be specified in compSymbolList and compNameList.
> In writeSBML (line 287)
  In writeCbModel (line 218)
Warning: Unknown compartment: C_ext.C_ext can be specified in compSymbolList and compNameList.
> In writeSBML (line 287)
  In writeCbModel (line 218)
Warning: Unknown compartment: C_m.C_m can be specified in compSymbolList and compNameList.
> In writeSBML (line 287)
  In writeCbModel (line 218)
Warning: Unknown compartment: C_p.C_p can be specified in compSymbolList and compNameList.
> In writeSBML (line 287)
  In writeCbModel (line 218)
Warning: Unknown compartment: C_v.C_v can be specified in compSymbolList and compNameList.
> In writeSBML (line 287)
  In writeCbModel (line 218)
Warning: Unknown compartment: C_x.C_x can be specified in compSymbolList and compNameList.
> In writeSBML (line 287)
  In writeCbModel (line 218)
Document written

i =

    'x8Control'

Warning: Unknown compartment: C_acc.C_acc can be specified in compSymbolList and compNameList.
> In writeSBML (line 287)
  In writeCbModel (line 218)
Warning: Unknown compartment: C_biomass.C_biomass can be specified in compSymbolList and compNameList.
> In writeSBML (line 287)
  In writeCbModel (line 218)
Warning: Unknown compartment: C_c.C_c can be specified in compSymbolList and compNameList.
> In writeSBML (line 287)
  In writeCbModel (line 218)
Warning: Unknown compartment: C_ext.C_ext can be specified in compSymbolList and compNameList.
> In writeSBML (line 287)
  In writeCbModel (line 218)
Warning: Unknown compartment: C_m.C_m can be specified in compSymbolList and compNameList.
> In writeSBML (line 287)
  In writeCbModel (line 218)
Warning: Unknown compartment: C_p.C_p can be specified in compSymbolList and compNameList.
> In writeSBML (line 287)
  In writeCbModel (line 218)
Warning: Unknown compartment: C_v.C_v can be specified in compSymbolList and compNameList.
> In writeSBML (line 287)
  In writeCbModel (line 218)
Warning: Unknown compartment: C_x.C_x can be specified in compSymbolList and compNameList.
> In writeSBML (line 287)
  In writeCbModel (line 218)
Document written

i =

    'x9Control'

Warning: Unknown compartment: C_acc.C_acc can be specified in compSymbolList and compNameList.
> In writeSBML (line 287)
  In writeCbModel (line 218)
Warning: Unknown compartment: C_biomass.C_biomass can be specified in compSymbolList and compNameList.
> In writeSBML (line 287)
  In writeCbModel (line 218)
Warning: Unknown compartment: C_c.C_c can be specified in compSymbolList and compNameList.
> In writeSBML (line 287)
  In writeCbModel (line 218)
Warning: Unknown compartment: C_ext.C_ext can be specified in compSymbolList and compNameList.
> In writeSBML (line 287)
  In writeCbModel (line 218)
Warning: Unknown compartment: C_m.C_m can be specified in compSymbolList and compNameList.
> In writeSBML (line 287)
  In writeCbModel (line 218)
Warning: Unknown compartment: C_p.C_p can be specified in compSymbolList and compNameList.
> In writeSBML (line 287)
  In writeCbModel (line 218)
Warning: Unknown compartment: C_v.C_v can be specified in compSymbolList and compNameList.
> In writeSBML (line 287)
  In writeCbModel (line 218)
Warning: Unknown compartment: C_x.C_x can be specified in compSymbolList and compNameList.
> In writeSBML (line 287)
  In writeCbModel (line 218)
Document written

i =

    'x10Control'

Warning: Unknown compartment: C_acc.C_acc can be specified in compSymbolList and compNameList.
> In writeSBML (line 287)
  In writeCbModel (line 218)
Warning: Unknown compartment: C_biomass.C_biomass can be specified in compSymbolList and compNameList.
> In writeSBML (line 287)
  In writeCbModel (line 218)
Warning: Unknown compartment: C_c.C_c can be specified in compSymbolList and compNameList.
> In writeSBML (line 287)
  In writeCbModel (line 218)
Warning: Unknown compartment: C_ext.C_ext can be specified in compSymbolList and compNameList.
> In writeSBML (line 287)
  In writeCbModel (line 218)
Warning: Unknown compartment: C_m.C_m can be specified in compSymbolList and compNameList.
> In writeSBML (line 287)
  In writeCbModel (line 218)
Warning: Unknown compartment: C_p.C_p can be specified in compSymbolList and compNameList.
> In writeSBML (line 287)
  In writeCbModel (line 218)
Warning: Unknown compartment: C_v.C_v can be specified in compSymbolList and compNameList.
> In writeSBML (line 287)
  In writeCbModel (line 218)
Warning: Unknown compartment: C_x.C_x can be specified in compSymbolList and compNameList.
> In writeSBML (line 287)
  In writeCbModel (line 218)
Document written

i =

    'x11Control'

Warning: Unknown compartment: C_acc.C_acc can be specified in compSymbolList and compNameList.
> In writeSBML (line 287)
  In writeCbModel (line 218)
Warning: Unknown compartment: C_biomass.C_biomass can be specified in compSymbolList and compNameList.
> In writeSBML (line 287)
  In writeCbModel (line 218)
Warning: Unknown compartment: C_c.C_c can be specified in compSymbolList and compNameList.
> In writeSBML (line 287)
  In writeCbModel (line 218)
Warning: Unknown compartment: C_ext.C_ext can be specified in compSymbolList and compNameList.
> In writeSBML (line 287)
  In writeCbModel (line 218)
Warning: Unknown compartment: C_m.C_m can be specified in compSymbolList and compNameList.
> In writeSBML (line 287)
  In writeCbModel (line 218)
Warning: Unknown compartment: C_p.C_p can be specified in compSymbolList and compNameList.
> In writeSBML (line 287)
  In writeCbModel (line 218)
Warning: Unknown compartment: C_v.C_v can be specified in compSymbolList and compNameList.
> In writeSBML (line 287)
  In writeCbModel (line 218)
Warning: Unknown compartment: C_x.C_x can be specified in compSymbolList and compNameList.
> In writeSBML (line 287)
  In writeCbModel (line 218)
Document written

i =

    'x12Control'

Warning: Unknown compartment: C_acc.C_acc can be specified in compSymbolList and compNameList.
> In writeSBML (line 287)
  In writeCbModel (line 218)
Warning: Unknown compartment: C_biomass.C_biomass can be specified in compSymbolList and compNameList.
> In writeSBML (line 287)
  In writeCbModel (line 218)
Warning: Unknown compartment: C_c.C_c can be specified in compSymbolList and compNameList.
> In writeSBML (line 287)
  In writeCbModel (line 218)
Warning: Unknown compartment: C_ext.C_ext can be specified in compSymbolList and compNameList.
> In writeSBML (line 287)
  In writeCbModel (line 218)
Warning: Unknown compartment: C_m.C_m can be specified in compSymbolList and compNameList.
> In writeSBML (line 287)
  In writeCbModel (line 218)
Warning: Unknown compartment: C_p.C_p can be specified in compSymbolList and compNameList.
> In writeSBML (line 287)
  In writeCbModel (line 218)
Warning: Unknown compartment: C_v.C_v can be specified in compSymbolList and compNameList.
> In writeSBML (line 287)
  In writeCbModel (line 218)
Warning: Unknown compartment: C_x.C_x can be specified in compSymbolList and compNameList.
> In writeSBML (line 287)
  In writeCbModel (line 218)
Document written

i =

    'x13Control'

Warning: Unknown compartment: C_acc.C_acc can be specified in compSymbolList and compNameList.
> In writeSBML (line 287)
  In writeCbModel (line 218)
Warning: Unknown compartment: C_biomass.C_biomass can be specified in compSymbolList and compNameList.
> In writeSBML (line 287)
  In writeCbModel (line 218)
Warning: Unknown compartment: C_c.C_c can be specified in compSymbolList and compNameList.
> In writeSBML (line 287)
  In writeCbModel (line 218)
Warning: Unknown compartment: C_ext.C_ext can be specified in compSymbolList and compNameList.
> In writeSBML (line 287)
  In writeCbModel (line 218)
Warning: Unknown compartment: C_m.C_m can be specified in compSymbolList and compNameList.
> In writeSBML (line 287)
  In writeCbModel (line 218)
Warning: Unknown compartment: C_p.C_p can be specified in compSymbolList and compNameList.
> In writeSBML (line 287)
  In writeCbModel (line 218)
Warning: Unknown compartment: C_v.C_v can be specified in compSymbolList and compNameList.
> In writeSBML (line 287)
  In writeCbModel (line 218)
Warning: Unknown compartment: C_x.C_x can be specified in compSymbolList and compNameList.
> In writeSBML (line 287)
  In writeCbModel (line 218)
Document written

i =

    'x1Drought'

Warning: Unknown compartment: C_acc.C_acc can be specified in compSymbolList and compNameList.
> In writeSBML (line 287)
  In writeCbModel (line 218)
Warning: Unknown compartment: C_biomass.C_biomass can be specified in compSymbolList and compNameList.
> In writeSBML (line 287)
  In writeCbModel (line 218)
Warning: Unknown compartment: C_c.C_c can be specified in compSymbolList and compNameList.
> In writeSBML (line 287)
  In writeCbModel (line 218)
Warning: Unknown compartment: C_ext.C_ext can be specified in compSymbolList and compNameList.
> In writeSBML (line 287)
  In writeCbModel (line 218)
Warning: Unknown compartment: C_m.C_m can be specified in compSymbolList and compNameList.
> In writeSBML (line 287)
  In writeCbModel (line 218)
Warning: Unknown compartment: C_p.C_p can be specified in compSymbolList and compNameList.
> In writeSBML (line 287)
  In writeCbModel (line 218)
Warning: Unknown compartment: C_v.C_v can be specified in compSymbolList and compNameList.
> In writeSBML (line 287)
  In writeCbModel (line 218)
Warning: Unknown compartment: C_x.C_x can be specified in compSymbolList and compNameList.
> In writeSBML (line 287)
  In writeCbModel (line 218)
Document written

i =

    'x2Drought'

Warning: Unknown compartment: C_acc.C_acc can be specified in compSymbolList and compNameList.
> In writeSBML (line 287)
  In writeCbModel (line 218)
Warning: Unknown compartment: C_biomass.C_biomass can be specified in compSymbolList and compNameList.
> In writeSBML (line 287)
  In writeCbModel (line 218)
Warning: Unknown compartment: C_c.C_c can be specified in compSymbolList and compNameList.
> In writeSBML (line 287)
  In writeCbModel (line 218)
Warning: Unknown compartment: C_ext.C_ext can be specified in compSymbolList and compNameList.
> In writeSBML (line 287)
  In writeCbModel (line 218)
Warning: Unknown compartment: C_m.C_m can be specified in compSymbolList and compNameList.
> In writeSBML (line 287)
  In writeCbModel (line 218)
Warning: Unknown compartment: C_p.C_p can be specified in compSymbolList and compNameList.
> In writeSBML (line 287)
  In writeCbModel (line 218)
Warning: Unknown compartment: C_v.C_v can be specified in compSymbolList and compNameList.
> In writeSBML (line 287)
  In writeCbModel (line 218)
Warning: Unknown compartment: C_x.C_x can be specified in compSymbolList and compNameList.
> In writeSBML (line 287)
  In writeCbModel (line 218)
Document written

i =

    'x3Drought'

Warning: Unknown compartment: C_acc.C_acc can be specified in compSymbolList and compNameList.
> In writeSBML (line 287)
  In writeCbModel (line 218)
Warning: Unknown compartment: C_biomass.C_biomass can be specified in compSymbolList and compNameList.
> In writeSBML (line 287)
  In writeCbModel (line 218)
Warning: Unknown compartment: C_c.C_c can be specified in compSymbolList and compNameList.
> In writeSBML (line 287)
  In writeCbModel (line 218)
Warning: Unknown compartment: C_ext.C_ext can be specified in compSymbolList and compNameList.
> In writeSBML (line 287)
  In writeCbModel (line 218)
Warning: Unknown compartment: C_m.C_m can be specified in compSymbolList and compNameList.
> In writeSBML (line 287)
  In writeCbModel (line 218)
Warning: Unknown compartment: C_p.C_p can be specified in compSymbolList and compNameList.
> In writeSBML (line 287)
  In writeCbModel (line 218)
Warning: Unknown compartment: C_v.C_v can be specified in compSymbolList and compNameList.
> In writeSBML (line 287)
  In writeCbModel (line 218)
Warning: Unknown compartment: C_x.C_x can be specified in compSymbolList and compNameList.
> In writeSBML (line 287)
  In writeCbModel (line 218)
Document written

i =

    'x4Drought'

Warning: Unknown compartment: C_acc.C_acc can be specified in compSymbolList and compNameList.
> In writeSBML (line 287)
  In writeCbModel (line 218)
Warning: Unknown compartment: C_biomass.C_biomass can be specified in compSymbolList and compNameList.
> In writeSBML (line 287)
  In writeCbModel (line 218)
Warning: Unknown compartment: C_c.C_c can be specified in compSymbolList and compNameList.
> In writeSBML (line 287)
  In writeCbModel (line 218)
Warning: Unknown compartment: C_ext.C_ext can be specified in compSymbolList and compNameList.
> In writeSBML (line 287)
  In writeCbModel (line 218)
Warning: Unknown compartment: C_m.C_m can be specified in compSymbolList and compNameList.
> In writeSBML (line 287)
  In writeCbModel (line 218)
Warning: Unknown compartment: C_p.C_p can be specified in compSymbolList and compNameList.
> In writeSBML (line 287)
  In writeCbModel (line 218)
Warning: Unknown compartment: C_v.C_v can be specified in compSymbolList and compNameList.
> In writeSBML (line 287)
  In writeCbModel (line 218)
Warning: Unknown compartment: C_x.C_x can be specified in compSymbolList and compNameList.
> In writeSBML (line 287)
  In writeCbModel (line 218)
Document written

i =

    'x5Drought'

Warning: Unknown compartment: C_acc.C_acc can be specified in compSymbolList and compNameList.
> In writeSBML (line 287)
  In writeCbModel (line 218)
Warning: Unknown compartment: C_biomass.C_biomass can be specified in compSymbolList and compNameList.
> In writeSBML (line 287)
  In writeCbModel (line 218)
Warning: Unknown compartment: C_c.C_c can be specified in compSymbolList and compNameList.
> In writeSBML (line 287)
  In writeCbModel (line 218)
Warning: Unknown compartment: C_ext.C_ext can be specified in compSymbolList and compNameList.
> In writeSBML (line 287)
  In writeCbModel (line 218)
Warning: Unknown compartment: C_m.C_m can be specified in compSymbolList and compNameList.
> In writeSBML (line 287)
  In writeCbModel (line 218)
Warning: Unknown compartment: C_p.C_p can be specified in compSymbolList and compNameList.
> In writeSBML (line 287)
  In writeCbModel (line 218)
Warning: Unknown compartment: C_v.C_v can be specified in compSymbolList and compNameList.
> In writeSBML (line 287)
  In writeCbModel (line 218)
Warning: Unknown compartment: C_x.C_x can be specified in compSymbolList and compNameList.
> In writeSBML (line 287)
  In writeCbModel (line 218)
Document written

i =

    'x6Drought'

Warning: Unknown compartment: C_acc.C_acc can be specified in compSymbolList and compNameList.
> In writeSBML (line 287)
  In writeCbModel (line 218)
Warning: Unknown compartment: C_biomass.C_biomass can be specified in compSymbolList and compNameList.
> In writeSBML (line 287)
  In writeCbModel (line 218)
Warning: Unknown compartment: C_c.C_c can be specified in compSymbolList and compNameList.
> In writeSBML (line 287)
  In writeCbModel (line 218)
Warning: Unknown compartment: C_ext.C_ext can be specified in compSymbolList and compNameList.
> In writeSBML (line 287)
  In writeCbModel (line 218)
Warning: Unknown compartment: C_m.C_m can be specified in compSymbolList and compNameList.
> In writeSBML (line 287)
  In writeCbModel (line 218)
Warning: Unknown compartment: C_p.C_p can be specified in compSymbolList and compNameList.
> In writeSBML (line 287)
  In writeCbModel (line 218)
Warning: Unknown compartment: C_v.C_v can be specified in compSymbolList and compNameList.
> In writeSBML (line 287)
  In writeCbModel (line 218)
Warning: Unknown compartment: C_x.C_x can be specified in compSymbolList and compNameList.
> In writeSBML (line 287)
  In writeCbModel (line 218)
Document written

i =

    'x7Drought'

Warning: Unknown compartment: C_acc.C_acc can be specified in compSymbolList and compNameList.
> In writeSBML (line 287)
  In writeCbModel (line 218)
Warning: Unknown compartment: C_biomass.C_biomass can be specified in compSymbolList and compNameList.
> In writeSBML (line 287)
  In writeCbModel (line 218)
Warning: Unknown compartment: C_c.C_c can be specified in compSymbolList and compNameList.
> In writeSBML (line 287)
  In writeCbModel (line 218)
Warning: Unknown compartment: C_ext.C_ext can be specified in compSymbolList and compNameList.
> In writeSBML (line 287)
  In writeCbModel (line 218)
Warning: Unknown compartment: C_m.C_m can be specified in compSymbolList and compNameList.
> In writeSBML (line 287)
  In writeCbModel (line 218)
Warning: Unknown compartment: C_p.C_p can be specified in compSymbolList and compNameList.
> In writeSBML (line 287)
  In writeCbModel (line 218)
Warning: Unknown compartment: C_v.C_v can be specified in compSymbolList and compNameList.
> In writeSBML (line 287)
  In writeCbModel (line 218)
Warning: Unknown compartment: C_x.C_x can be specified in compSymbolList and compNameList.
> In writeSBML (line 287)
  In writeCbModel (line 218)
Document written

i =

    'x8Drought'

Warning: Unknown compartment: C_acc.C_acc can be specified in compSymbolList and compNameList.
> In writeSBML (line 287)
  In writeCbModel (line 218)
Warning: Unknown compartment: C_biomass.C_biomass can be specified in compSymbolList and compNameList.
> In writeSBML (line 287)
  In writeCbModel (line 218)
Warning: Unknown compartment: C_c.C_c can be specified in compSymbolList and compNameList.
> In writeSBML (line 287)
  In writeCbModel (line 218)
Warning: Unknown compartment: C_ext.C_ext can be specified in compSymbolList and compNameList.
> In writeSBML (line 287)
  In writeCbModel (line 218)
Warning: Unknown compartment: C_m.C_m can be specified in compSymbolList and compNameList.
> In writeSBML (line 287)
  In writeCbModel (line 218)
Warning: Unknown compartment: C_p.C_p can be specified in compSymbolList and compNameList.
> In writeSBML (line 287)
  In writeCbModel (line 218)
Warning: Unknown compartment: C_v.C_v can be specified in compSymbolList and compNameList.
> In writeSBML (line 287)
  In writeCbModel (line 218)
Warning: Unknown compartment: C_x.C_x can be specified in compSymbolList and compNameList.
> In writeSBML (line 287)
  In writeCbModel (line 218)
Document written

i =

    'x9Drought'

Warning: Unknown compartment: C_acc.C_acc can be specified in compSymbolList and compNameList.
> In writeSBML (line 287)
  In writeCbModel (line 218)
Warning: Unknown compartment: C_biomass.C_biomass can be specified in compSymbolList and compNameList.
> In writeSBML (line 287)
  In writeCbModel (line 218)
Warning: Unknown compartment: C_c.C_c can be specified in compSymbolList and compNameList.
> In writeSBML (line 287)
  In writeCbModel (line 218)
Warning: Unknown compartment: C_ext.C_ext can be specified in compSymbolList and compNameList.
> In writeSBML (line 287)
  In writeCbModel (line 218)
Warning: Unknown compartment: C_m.C_m can be specified in compSymbolList and compNameList.
> In writeSBML (line 287)
  In writeCbModel (line 218)
Warning: Unknown compartment: C_p.C_p can be specified in compSymbolList and compNameList.
> In writeSBML (line 287)
  In writeCbModel (line 218)
Warning: Unknown compartment: C_v.C_v can be specified in compSymbolList and compNameList.
> In writeSBML (line 287)
  In writeCbModel (line 218)
Warning: Unknown compartment: C_x.C_x can be specified in compSymbolList and compNameList.
> In writeSBML (line 287)
  In writeCbModel (line 218)
Document written

i =

    'x10Drought'

Warning: Unknown compartment: C_acc.C_acc can be specified in compSymbolList and compNameList.
> In writeSBML (line 287)
  In writeCbModel (line 218)
Warning: Unknown compartment: C_biomass.C_biomass can be specified in compSymbolList and compNameList.
> In writeSBML (line 287)
  In writeCbModel (line 218)
Warning: Unknown compartment: C_c.C_c can be specified in compSymbolList and compNameList.
> In writeSBML (line 287)
  In writeCbModel (line 218)
Warning: Unknown compartment: C_ext.C_ext can be specified in compSymbolList and compNameList.
> In writeSBML (line 287)
  In writeCbModel (line 218)
Warning: Unknown compartment: C_m.C_m can be specified in compSymbolList and compNameList.
> In writeSBML (line 287)
  In writeCbModel (line 218)
Warning: Unknown compartment: C_p.C_p can be specified in compSymbolList and compNameList.
> In writeSBML (line 287)
  In writeCbModel (line 218)
Warning: Unknown compartment: C_v.C_v can be specified in compSymbolList and compNameList.
> In writeSBML (line 287)
  In writeCbModel (line 218)
Warning: Unknown compartment: C_x.C_x can be specified in compSymbolList and compNameList.
> In writeSBML (line 287)
  In writeCbModel (line 218)
Document written

i =

    'x11Drought'

Warning: Unknown compartment: C_acc.C_acc can be specified in compSymbolList and compNameList.
> In writeSBML (line 287)
  In writeCbModel (line 218)
Warning: Unknown compartment: C_biomass.C_biomass can be specified in compSymbolList and compNameList.
> In writeSBML (line 287)
  In writeCbModel (line 218)
Warning: Unknown compartment: C_c.C_c can be specified in compSymbolList and compNameList.
> In writeSBML (line 287)
  In writeCbModel (line 218)
Warning: Unknown compartment: C_ext.C_ext can be specified in compSymbolList and compNameList.
> In writeSBML (line 287)
  In writeCbModel (line 218)
Warning: Unknown compartment: C_m.C_m can be specified in compSymbolList and compNameList.
> In writeSBML (line 287)
  In writeCbModel (line 218)
Warning: Unknown compartment: C_p.C_p can be specified in compSymbolList and compNameList.
> In writeSBML (line 287)
  In writeCbModel (line 218)
Warning: Unknown compartment: C_v.C_v can be specified in compSymbolList and compNameList.
> In writeSBML (line 287)
  In writeCbModel (line 218)
Warning: Unknown compartment: C_x.C_x can be specified in compSymbolList and compNameList.
> In writeSBML (line 287)
  In writeCbModel (line 218)
Document written

i =

    'x12Drought'

Warning: Unknown compartment: C_acc.C_acc can be specified in compSymbolList and compNameList.
> In writeSBML (line 287)
  In writeCbModel (line 218)
Warning: Unknown compartment: C_biomass.C_biomass can be specified in compSymbolList and compNameList.
> In writeSBML (line 287)
  In writeCbModel (line 218)
Warning: Unknown compartment: C_c.C_c can be specified in compSymbolList and compNameList.
> In writeSBML (line 287)
  In writeCbModel (line 218)
Warning: Unknown compartment: C_ext.C_ext can be specified in compSymbolList and compNameList.
> In writeSBML (line 287)
  In writeCbModel (line 218)
Warning: Unknown compartment: C_m.C_m can be specified in compSymbolList and compNameList.
> In writeSBML (line 287)
  In writeCbModel (line 218)
Warning: Unknown compartment: C_p.C_p can be specified in compSymbolList and compNameList.
> In writeSBML (line 287)
  In writeCbModel (line 218)
Warning: Unknown compartment: C_v.C_v can be specified in compSymbolList and compNameList.
> In writeSBML (line 287)
  In writeCbModel (line 218)
Warning: Unknown compartment: C_x.C_x can be specified in compSymbolList and compNameList.
> In writeSBML (line 287)
  In writeCbModel (line 218)
Document written

i =

    'x13Drought'

Warning: Unknown compartment: C_acc.C_acc can be specified in compSymbolList and compNameList.
> In writeSBML (line 287)
  In writeCbModel (line 218)
Warning: Unknown compartment: C_biomass.C_biomass can be specified in compSymbolList and compNameList.
> In writeSBML (line 287)
  In writeCbModel (line 218)
Warning: Unknown compartment: C_c.C_c can be specified in compSymbolList and compNameList.
> In writeSBML (line 287)
  In writeCbModel (line 218)
Warning: Unknown compartment: C_ext.C_ext can be specified in compSymbolList and compNameList.
> In writeSBML (line 287)
  In writeCbModel (line 218)
Warning: Unknown compartment: C_m.C_m can be specified in compSymbolList and compNameList.
> In writeSBML (line 287)
  In writeCbModel (line 218)
Warning: Unknown compartment: C_p.C_p can be specified in compSymbolList and compNameList.
> In writeSBML (line 287)
  In writeCbModel (line 218)
Warning: Unknown compartment: C_v.C_v can be specified in compSymbolList and compNameList.
> In writeSBML (line 287)
  In writeCbModel (line 218)
Warning: Unknown compartment: C_x.C_x can be specified in compSymbolList and compNameList.
> In writeSBML (line 287)
  In writeCbModel (line 218)
Document written
```

In [7]:

```
summaryT
```

```
summaryT =

  27x4 table

     condition      mets    rxns     FBA  
    ____________    ____    ____    ______

    'x0Zero'        1348    1173    124.65
    'x1Control'     1345    1171    124.65
    'x2Control'     1351    1173    124.65
    'x3Control'     1345    1157    124.65
    'x4Control'     1358    1182    124.65
    'x5Control'     1358    1184    124.65
    'x6Control'     1340    1159    124.65
    'x7Control'     1331    1161    124.65
    'x8Control'     1356    1179    124.65
    'x9Control'     1357    1178    124.65
    'x10Control'    1348    1157    124.65
    'x11Control'    1341    1155    124.65
    'x12Control'    1329    1133    129.14
    'x13Control'    1330    1144    129.14
    'x1Drought'     1341    1157    124.65
    'x2Drought'     1345    1163    124.65
    'x3Drought'     1357    1177    124.65
    'x4Drought'     1345    1168    124.65
    'x5Drought'     1334    1151    124.65
    'x6Drought'     1339    1161    124.65
    'x7Drought'     1334    1162    124.65
    'x8Drought'     1331    1152    124.65
    'x9Drought'     1352    1180    124.65
    'x10Drought'    1348    1183    124.65
    'x11Drought'    1330    1152    124.65
    'x12Drought'    1322    1143    124.65
    'x13Drought'    1337    1165    124.65
```

In [ ]:

```

```
