# Supplementary material for: Drought Stress Responses in Context-Specific Genome-Scale Metabolic Models of Arabidopsis thaliana
Source: Metabolites. 2020 Apr 18;10(4):159. doi: 10.3390/metabo10040159 (PMC7241242; doi:10.3390/metabo10040159)
Supplement: Supplementary file 1 [file metabolites-10-00159-s001.zip › metabolites-736140-SP/Supplementary figures.pdf]

metabolites

Supplementary Figures

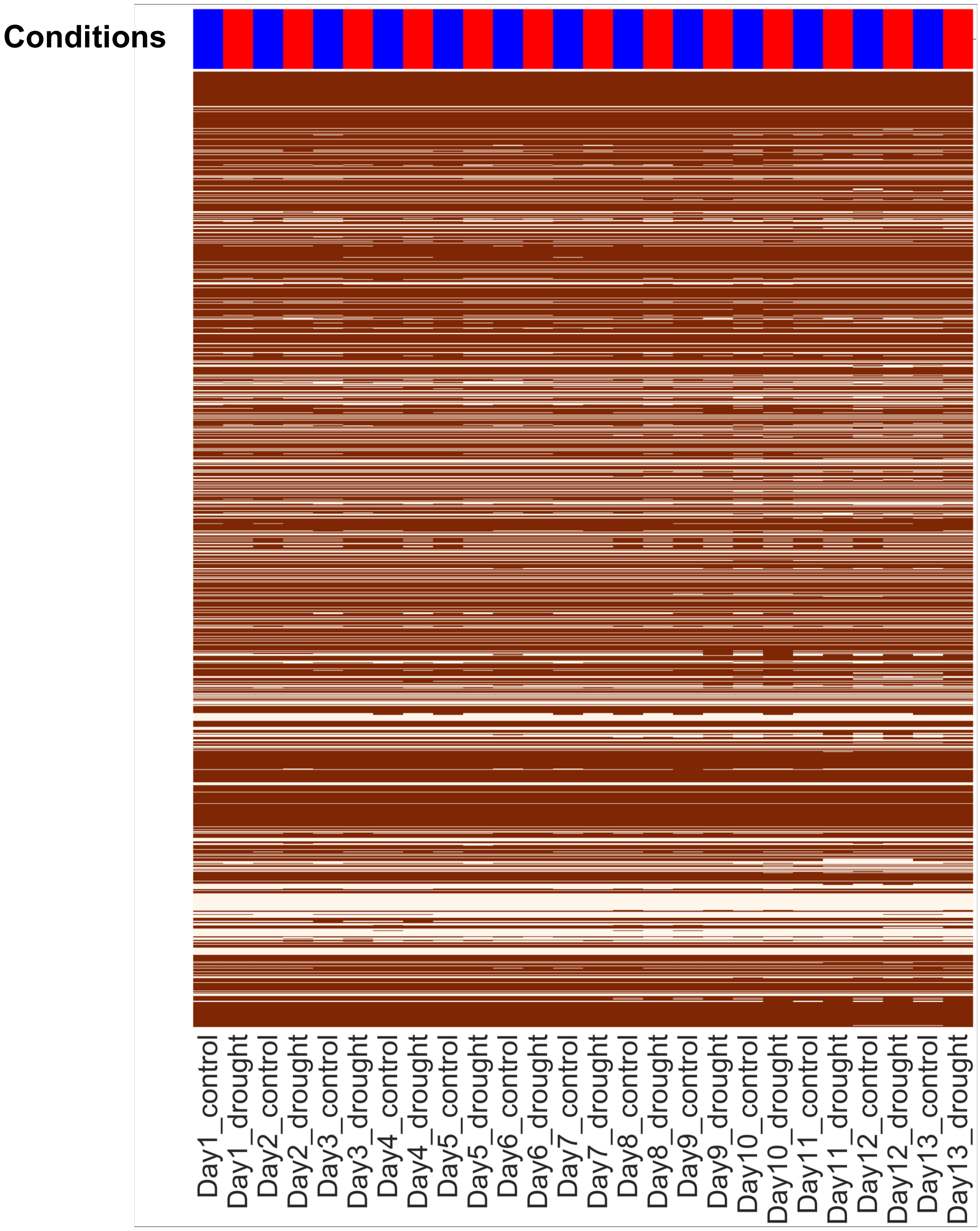

Figure S1. Heatmap depicting present or absent reactions in 26 context-specific GEMs. The rows represent 1601 reactions in the global GEM (Supplementary data 1, AraGEMconstraintFree.xls). The color indicates presence (brown) or absence (white) in each context-specific GEM. Conditions indicate control (blue) or drought (red).

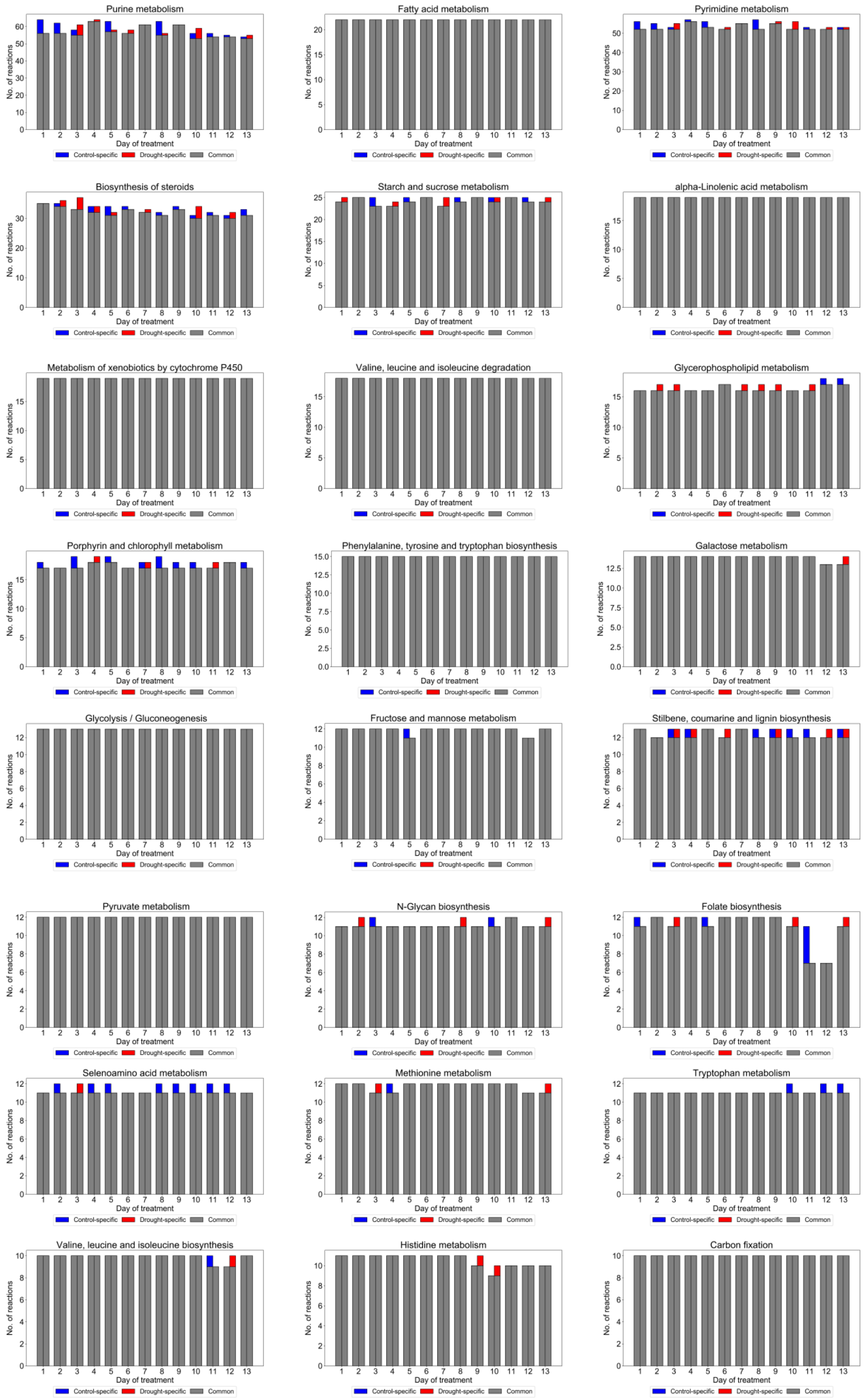

Figure S2. The number of reactions in respective subsystems in each GEM. Only the subsystems which contain more than 10 reactions are shown. The left and right bars in a day represent the number in control- and drought-GEMs, respectively. The control-specific, drought-specific, and common reactions are displayed in blue, red, and grey, respectively.

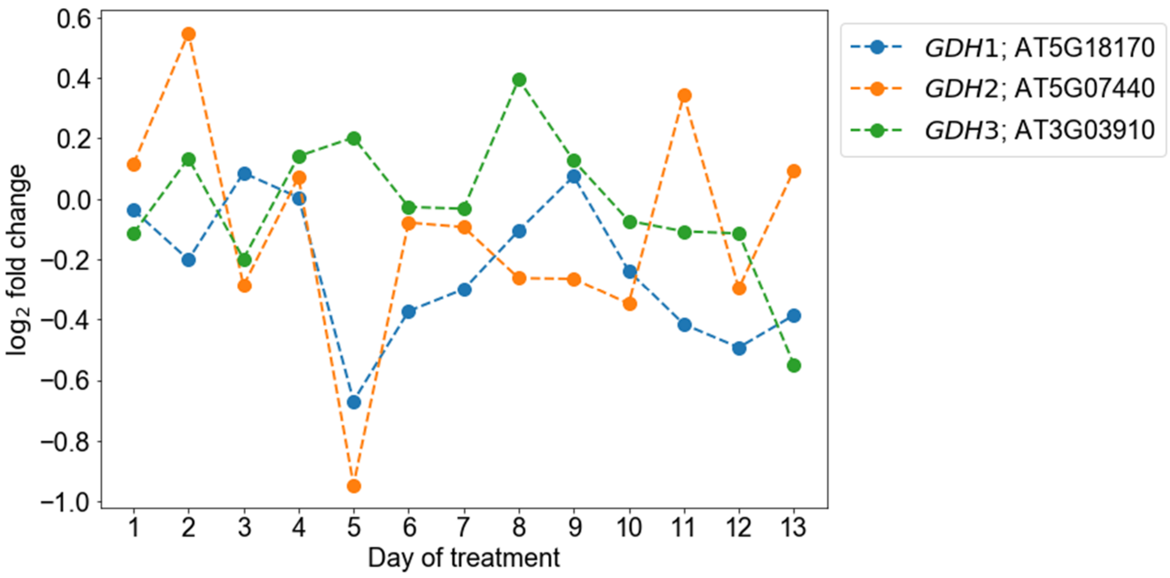

Figure S3. Changes in glutamate dehydrogenase gene expression under drought stress in Arabidopsis. Log_2 fold change of average gene expression levels of glutamate dehydrogenase genes *GDH1*, *GDH2* and *GDH3* are shown. The data was retrieved from Bechtold et al (2016) [16].

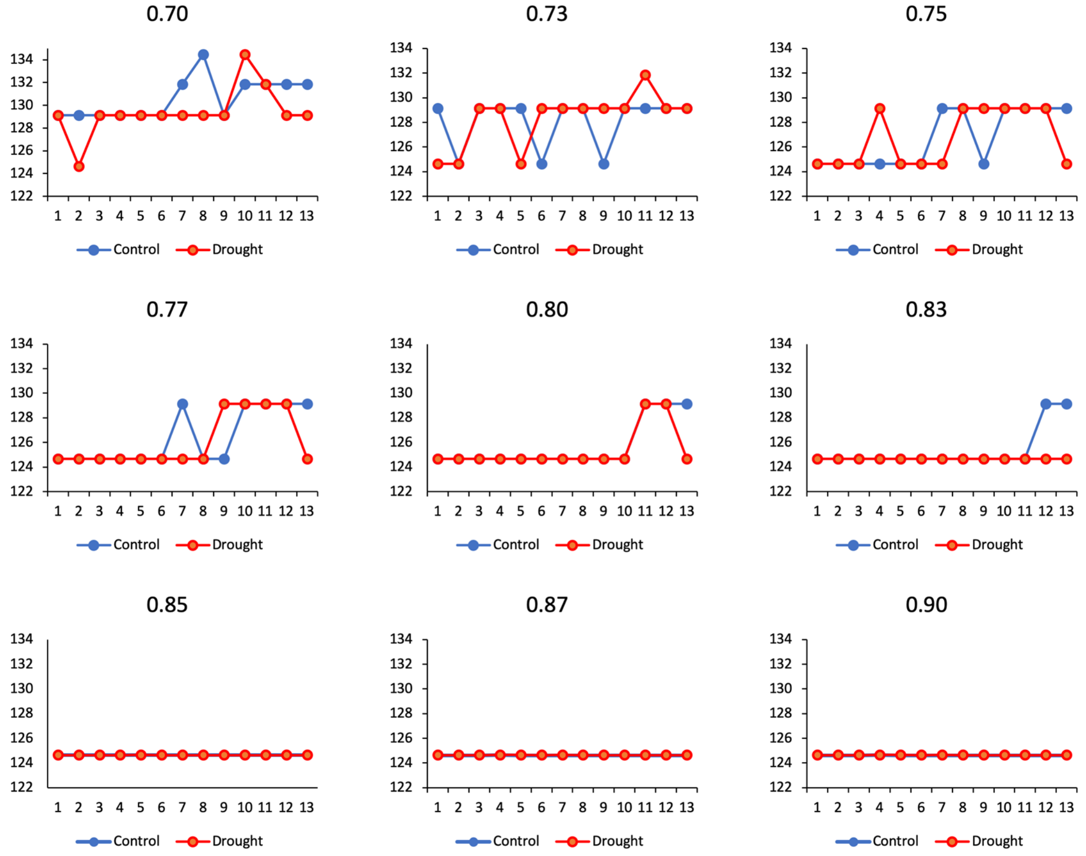

Figure S4. Effect of threshold setting on the time-dependent pattern of biomass production rate of 26 context-specific GEMs.
