# Supplementary material for: Drought Stress Responses in Context-Specific Genome-Scale Metabolic Models of Arabidopsis thaliana
Source: Metabolites. 2020 Apr 18;10(4):159. doi: 10.3390/metabo10040159 (PMC7241242; doi:10.3390/metabo10040159)
Supplement: Supplementary file 1 [file metabolites-10-00159-s001.zip › metabolites-736140-SP/Supplementary Table 1 Reactions involved in drought metabolic adaptation in terms of biomass production.pdf]

Supplementary Table 1 Reactions involved in drought metabolic adaptation in terms of biomass production

Reaction ID	Compartment	Reaction name	EC	Reference information	Reference
R00243_c	cytoplasm	glutamate dehydrogenase	1.4.1.2; 1.4.1.3	Overexpression of *E.coli* gdhA enhanced drought tolerance and biomass production in tobacco	Mungur et al. (2006) [1]
				Overexpression of *E.coli* gdhA enhanced drought tolerance and biomass production in maize	Lightfoot et al. (2007) [2]
R01070_p	plastid	fructose-bisphosphate aldolase	4.1.2.13	Eight fructose-bisphosphate aldolase family genes in *Arabidopsis* showed different expression patterns in response to abiotic stresses including drought	Lu et al. (2012) [3]
				Fructose-bisphosphate aldolase family genes in wheat (*Triticum aestivum* L.) were characterized and some genes involved in responses to various abiotic stresses including drought	Lv et al. (2017) [4]
				Expression of fructose-1,6-bisphosphate aldolase gene was induced by high-salinity and drought in shoreline purslane mangrove (*Sesuvium portulacastrum*)	Fan et al. (2009) [5]
R00588_x	peroxisome	serine-glyoxylate aminotransferase	2.6.1.45	Serine-glyoxylate transaminase plays an important role in photorespiration during drought stress in barley	Wingler et al. (1999); Wingler et al. (2000) [6,7]
R01388_x	peroxisome	hydroxypyruvate reductase	1.1.1.81	Mutation in the hydroxypyruvate reductase 1 gene of *Arabidopsis* enhanced the susceptibility to drought stress	Li et al. (2015) [8]

Reference:

1. Mungur, R.; Wood, A.J.; Lightfoot, D.A. Water potential is maintained during water deficit in Nicotiana tabacum expressing the Escherichia coli glutamate dehydrogenase gene. *Plant Growth Regul.* **2006**, *50*, 231–238, doi:10.1007/s10725-006-9140-4.
2. Lightfoot, D.A.; Mungur, R.; Ameziane, R.; Nolte, S.; Long, L.; Bernhard, K.; Colter, A.; Jones, K.; Iqbal, M.J.; Varsa, E.; et al. Improved drought tolerance of transgenic Zea mays plants that express the glutamate dehydrogenase gene (gdhA) of E-coli. *Euphytica* **2007**, *156*, 103–116, doi:10.1007/s10681-007-9357-y.
3. Lu, W.; Tang, X.; Huo, Y.; Xu, R.; Qi, S.; Huang, J.; Zheng, C.; Wu, C.A. Identification and characterization of fructose 1,6-bisphosphate aldolase genes in Arabidopsis reveal a gene family with diverse responses to abiotic stresses. *Gene* **2012**, *503*, 65–74, doi:10.1016/j.gene.2012.04.042.
4. Lv, G.Y.; Guo, X.G.; Xie, L.P.; Xie, C.G.; Zhang, X.H.; Yang, Y.; Xiao, L.; Tang, Y.Y.; Pan, X.L.; Guo, A.G.; et al. Molecular characterization, gene evolution, and expression analysis of the fructose-1, 6-bisphosphate aldolase (FBA) gene family in wheat *(Triticum aestivum L). Front. Plant Sci.* **2017**, *8*, 1030, doi:10.3389/fpls.2017.01030.

5. Fan, W.; Zhang, Z.; Zhang, Y. Cloning and molecular characterization of fructose-1,6-bisphosphate aldolase gene regulated by high-salinity and drought in *Sesuvium portulacastrum*. *Plant Cell Rep.* **2009**, *28*, 975–984, doi:10.1007/s00299-009-0702-6.
6. Wingler, A.; Quick, W.P.; Bungard, R.A.; Bailey, K.J.; Lea, P.J.; Leegood, R.C. The role of photorespiration during drought stress: An analysis utilizing barley mutants with reduced activities of photorespiratory enzymes. *Plant Cell Environ.* **1999**, *22*, 361–373, doi:10.1046/j.1365-3040.1999.00410.x.
7. Wingler, A.; Lea, P.J.; Quick, W.P.; Leegood, R.C. Photorespiration: Metabolic pathways and their role in stress protection. *Philos Trans. R. Soc. Lond. B Biol. Sci.* **2000**, *355*, 1517–1529, doi:10.1098/rstb.2000.0712.
8. Li, J.; Hu, J. Using co-expression analysis and stress-based screens to uncover Arabidopsis peroxisomal proteins involved in drought response. *PLoS ONE* **2015**, *10*, e0137762, doi:10.1371/journal.pone.0137762.
